# Influence of resection distance on vulvar cancer relapse: a retrospective analysis

**DOI:** 10.1007/s00404-025-08046-y

**Published:** 2025-05-21

**Authors:** Jan-Philipp Cieslik, Annika Sophia Beerbaum, Tanja Fehm, Monika Hampl

**Affiliations:** 1 Department of Gynecology and Obstetrics, University Hospital Düsseldorf, Moorenstraße 5, 40225 Düsseldorf, Germany; 2 Center for Integrated Oncology (CIO Aachen, Bonn, Cologne, Düsseldorf), Düsseldorf, Germany

**Keywords:** Vulvar squamous cell carcinoma, Resection margin, Disease-free survival, Lichen sclerosus, Lymph node metastasis, Local recurrence

## Abstract

**Objective:**

This study evaluates the effect of resection margin distance on disease-free survival (DFS) and (local) recurrence rates in patients with vulvar squamous cell carcinoma (SCC) while assessing the impact of associated factors such as lichen sclerosus (LS) and lymph node metastasis.

**Methods:**

A retrospective single-center analysis was conducted on 150 patients treated for vulvar SCC between 2004 and 2014 at University Hospital Düsseldorf. Univariate and multivariate regression analyses were performed to evaluate the impact of clinical and pathological factors on DFS. Additionally, a literature review was conducted to summarize existing evidence on resection margins.

**Results:**

The findings suggest that a resection margin exceeding 8 mm does not significantly improve DFS (HR 1.14, CI 1.01–1.28, *p *= 0.029). LS was significantly associated with recurrence (HR 2.36, CI 1.13–4.91, *p *= 0.02) and reduced DFS. Univariate analysis identified lymph node metastasis as a significant predictor of DFS; however, this association was not retained in multivariate analysis.

**Conclusion:**

Although current guidelines advocate for resection margins >8 mm, our findings suggest that smaller margins may be acceptable in selected patients, particularly those without LS and tumors located near critical structures (e.g., the anus, clitoris, or urethra). These considerations should inform personalized treatment strategies and follow-up care.

**Supplementary Information:**

The online version contains supplementary material available at 10.1007/s00404-025-08046-y.

## What does this study add to the clinical work

**Table:** 

Our study suggests that resection margins > 8 mm may not significantly improve disease-free survival in vulvar SCC and that factors like lichen sclerosus and lymph node metastasis are stronger predictors of recurrence. Personalized surgical approaches with closer follow-up may be safely considered in select low-risk patients.	

## Introduction

Vulvar cancer is characterized by the malignant transformation of the vulvar epithelium. It is the fourth most common gynecological cancer worldwide, with an annual incidence exceeding 47,000 cases and a mortality rate surpassing 18,000 deaths in 2022 [1]. The National Comprehensive Cancer Network (NCCN) guidelines recommend a resection margin of at least 1–2 cm, based on four studies published between 1990 and 2007 [2]. In 1990, Heaps et al. published data from 135 patients and found that none of the 91 patients with resection margins over 8 mm had a local recurrence, while 51% of the patients with smaller resection margins demonstrated local recurrence (*p* $<$ 0.0001). Heaps et al. did not report additional clinical data, such as the presence of lichen sclerosus (LS) [3]. Next, in 2003, Rouzier et al. described a cohort of 215 patients [4]. Most of the patients (171) achieved a resection margin of over 1 cm, which was correlated with improved disease-specific survival (DFS) (*p* $<$ 0.001) and better local relapse-free survival (*p* $<$ 0.001) [4]. Interestingly, LS was not significantly associated with either outcome [5]. LS is a chronic inflammatory skin disease well described to increase the risk of vulvar cancer [5, 6]. Further, de Hullu et al. analyzed 253 patients between 1982 and 1997 [7]. The authors compared wide local excision with radical vulvectomy. The local recurrence rate was significantly elevated in the wide local excision group when tumor-free margins were less than 8 mm (22.5% vs. 0% *p *= 0.002). The authors did not report concomitant LS disease. Another cited study by the NCCN guidelines is the 2007 study by Chan et al., which reviewed 90 patients from 1984 to 2002 and found that no patients with a resection margin over 8 mm had local recurrence, while 23% of patients with less than 8 mm demonstrated a local recurrence (*p *= 0.002) [8]. The study did not comment on the influence of LS on local recurrence. While the NCCN guidelines suggest a correlation between resection margin distance and survival in vulvar cancer, this remains a subject of ongoing debate in clinical practice. This study critically evaluates existing literature and presents data from a single-center experience to highlight key aspects of this ongoing discussion.

## Materials and methods

### Literature review

To evaluate the current evidence on resection margins in vulvar cancer, we conducted a literature review using PubMed, employing MeSH terms, publication type restrictions, and specific search terms for titles and abstracts (Table S1). The query parameters aimed to find original research papers on resection distance in vulvar cancer, excluding meta-analysis and case reports. We followed the PRISMA guidelines for systematic reviews [9]. A clinician screened the abstracts using the Rayyan platform, without employing artificial intelligence add-ons. Afterwards, full-text articles were retrieved and screened. All included articles were then summarized by clinicians.

### Patient cohort

Our study includes 150 patients with squamous cell carcinoma (SCC) of the vulva who were treated at the University Hospital Düsseldorf, Germany, between 02/2004 and 12/2014. The majority of patients (140) underwent surgery at our institution, while 10 were referred following primary surgery at another hospital. In all cases, a histopathological examination was conducted, providing information on the margin size of resection (in mm), confirming the entity of vulvar SCC, and detailing the results of histopathological inguinal lymph node staging and postoperative follow-up findings. The patients with other vulvar neoplasms, including malignant melanomas, adenocarcinomas, Paget’s disease, basal cell carcinomas, and sarcomas, as well as carcinoma in situ (CIS) and microinvasive carcinomas (pT1a), were excluded. Only macroinvasive SCCs of the vulva (≥ pT1b) were included.

### Data acquisition and study design

It was a single-center, retrospective study conducted at the Department of Gynecology and Obstetrics of the University Hospital in Düsseldorf. An ethical approval was issued by the ethics committee of the faculty of medicine at Heinrich Heine University Düsseldorf (study number 5433). The clinical data of the patients, the histopathological characteristics of the diagnosed vulvar carcinomas, and information on therapies and follow-ups were extracted from patient records, the clinical information system, and a specific questionnaire. A tailored questionnaire was distributed to all patients to ensure comprehensive data collection, particularly concerning follow-up, and potential recurrence details.

### Surgical management

All patients included in this analysis underwent surgical treatment for vulvar carcinoma, as well as invasive staging of inguinal lymph nodes. Lymph node staging was primarily performed through sentinel lymph node biopsy (tumor diameter < 4 cm). Inguinofemoral lymph node dissection was performed in cases of suspected metastasis or evidence of metastatic disease in frozen sections. In cases where evidence of metastasis was first reported in the final histology, a follow-up surgery was performed. Professor Monika Hampl primarily conducted surgical therapy for vulvar carcinoma at the University Hospital Düsseldorf.

Depending on the extent of the findings, vulvar carcinomas were resected using radical local excision, hemivulvectomy, or complete vulvectomy with a three-incision technique. For small tumors near critical structures (such as the clitoris, urethra, or anus), the patients were offered tumor resection with reduced radicality in accordance with guidelines, omitting the conventional margin of > 8 mm. Margin distances of 3–4 mm were tolerated to preserve organs (especially the clitoris/urethra). This approach was primarily considered for younger patients without risk factors, demonstrating good adherence to follow-up, and presenting with small, localized carcinomas in the anterior fourchette. Detailed preoperative counseling regarding the potential increased risk of recurrence was provided, as well as the need for regular follow-up every 3 months. This strategy enabled the documentation of several cases with reduced resection margins. This study investigates whether these reduced margin distances in our patient population correlate with an increased recurrence rate and/or reduced disease-free survival.

### Determination of resection margin distance

The formalin-fixed and paraffin-embedded tumor specimens underwent complete microscopic examination postoperatively at the Pathological Institute of the Heinrich Heine University Düsseldorf following hematoxylin-eosin staining. The analysis was conducted by two clinicians using the Wilkinson method. The respective histological findings, including pathological tumor size, depth of infiltration, and the minimum tumor-free margin distance (in mm) from the specimen edge (at four points), were subsequently recorded in the clinical information system of the Department of Gynecology at University Hospital Düsseldorf.

### Postoperative course and follow-up

A structured follow-up schedule was implemented after surgery to facilitate the early detection of recurrence. The patients were advised to undergo follow-up examinations every 3 months for the first 2 years postoperatively and then every 6 months for the subsequent three years, either at the Department of Gynecology at University Hospital Düsseldorf or with their primary gynecologist. The patients were encouraged to seek medical attention if they experienced abnormalities or complications between these intervals.

### Questionnaire

A questionnaire was developed and sent to all patients to gather additional information, particularly regarding potential recurrence and the timing of diagnosis. The time of the last documented follow-up examination or response to the questionnaire marked the end of the observation period. The questionnaire also covered general aspects (occupation, nationality, and name of gynecologist), gynecological history (LS, history of HPV infections, gynecological infections, follow-up intervals, and gynecological carcinomas), original presenting symptoms of vulvar carcinoma (burning and pain), quality of life after treatment compared to the preoperative situation, and any postoperative restrictions (especially leg edema, incontinence, and sexual dysfunction).

### Statistical analysis

The statistical language R (version 4.2.2) was used for all analyses. The influence of clinical-pathological prognostic factors (independent variables) on recurrence rates (dependent variables) was analyzed in univariate and multivariate regressions. The impact of the recurrence location was determined via generalized linear models (GLM). The influence of variables on DFS was determined univariately using the Kaplan–Meier estimator and multivariately using Cox regression. All p-values were two-tailed, and the significance level was set at an alpha level of $\le$ 0.05.

## Results

### Literature review

Based on the systematic search parameters described earlier, we retrieved 60 abstracts, which were subsequently screened (Fig. S1, Table S1). Of those abstracts, 38 were excluded and the remaining 22 reports were retrieved as full text (Fig. S1). After full-text screening, 18 articles remained, which were summarized (Fig. S1).

In total, 12 of the 18 reports describe no significant association between 8 mm margin distance and survival or recurrence (Table 1) [10–21]. A correlation between recurrence and > 8 mm margin distance was found in three studies [3, 22, 23]. Further, two studies described a significant correlation between >8 mm margin distance and OS/DSS [8, 24]. One study only found a significant correlation between OS and positive margins (no margin at all) [25].

**Table 1:** Literature review (CT = chemotherapy, ed = epithelial disorder, LN = lymph node, LVSI = lymphovascular space invasion, LRFS = local recurrence free survival, RT = radiotherapy, ^**+**^ = median, * = mean)

*N* = Number of patients *F* = Follow-up	Collective	Outcome Recurrence rate	Findings
2023 [10] Germany Taran *n* = 128 *F* = 78^+^	SCC, node-negative, no adjuvant treatment	DFS, OS RR = 30.47%	No significant difference in DFS/OS based on margins;
2021 [25] Japan Nomura *n* = 34 *F* = 70^*^	SCC, no distant metastasis	LRFS, OS RR = 41.18%	Shorter OS with positive margins (*p* = 0.0013) Shorter OS with adj. RT (*p* = 0.0044) No difference between margin cutoffs
2020 [11] Australia Barlow *n* = 345 *F* = 93^+^	SCC	PFS, DSS RR = 22.61%	No difference in local recurrence (< 8 mm vs > 8 mm *p* = 0.65) Difference in local recurrence for 5 mm cutoff (*p* = 0.02) Difference when stratified by local and remote vulvar site (< 8 mm more likely to be local while > 8 mm more likely to be distant *p* < 0.001)
2020 [24] Italy Pecorino *n* = 118 *F* = 84^+^	SCC	OS, DSS RR = 24.58%	Margins < 8 mm significantly decreased DSS and OS (*p* = 0.015; *p* = 0.001)
2019 [12] France Raimond *n* = 112 *F* = 25^*^	SCC	DFS RR = 26.79%	No significant difference in survival between cutoffs
2019 [13] Netherlands Te Grootenhuis *n* = 287 *F* = 80^+^	SCC	LRFS RR = 42.51%	No significant difference in survival between cutoffs dVIN and/or LSA in margin influenced recurrence (*p* < 0.001; *p* = 0.04)
2018 [14] Netherlands Pleunis *n* = 167 *F* = 40^+^	SCC, FIGO IB-IIIC	LRFS RR = 21.56%	no significant difference in survival between cutoffs LSA was associated with LRFS (*p* < 0.01)
2018 [15] Italy Micheletti *n* = 114 *F* = 80^+^	SCC, FIGO IB/II	OS, DSS, LRFS RR = 40.35%	8 mm cutoff was not significant for OS or DSS OS significantly lower < 5 mm (*p* = 0.002) DSS significantly lower < 5 mm (*p* = 0.033)
2018 [22] Turkey Arvas *n* = 107 *F* = 69^+^	SCC	LRFS RR = 45.79%	Local recurrence significantly higher in < 2 mm and < 8 mm (*p* = 0.008) Total recurrence significantly higher in < 2 mm and < 8 mm (*p* = 0.0001)
2018 [23] Czech Republic Minar *n* = 47 *F* = N/A (range 4–105)	SCC	DFS, OS RR = 27.66%	Local recurrence significantly higher in < 8 mm (*p* = 0.003) Diameter, Depth, LVSI, Midline involement, metastasis, stage significant for recurrence
2016 [16] Germany Woelber *n* = 289 *F* = 35^+^	SCC, IB or higher, R0	LRFS, DFS RR = 21.80%	Margin not significantly associated with local recurrence
2015 [17] Brazil Baiocchi *n* = 205 *F* = 36^+^	SCC	DFS, LRFS RR = 38.05%	Margin not significantly associated with local recurrence or DFS (*p* = 0.98; *p* = 0.94)
2013 [18] Spain Iacoponi *n* = 87 *F* = 32^*^	SCC	DFS RR = 43.68%	Margin not significantly associated with local recurrence (*p* = 0.5 comparing < 8 mm and > 8 mm; *p* = 0.09 comparing < 15 mm and > 15 mm)
2011 [19] Germany Woelber *n* = 102 *F* = 31^+^	SCC	DFS RR = 15.69%	Margin not significantly associated with local recurrence (*p* = 0.388)
2010 [20] Netherlands Groenen *n* = 93 *F* = 31^+^	SCC	LRFS RR = 21.69%	Margin not significantly associated with local recurrence
2007 [8] USA Chan *n* = 90 *F* = 58^+^	SCC	DSS RR = 16.67%	DSS significantly reduced when margin < 8 mm (*p* = 0.003)
1990 [3] USA Heaps *n* = 135 *F* = N/A	SCC	LRFS RR = 15.56%	Only patients with margin < 8 mm developed a recurrence (*p* < 0.0001)
1990 [21] USA Burke *n* = 32 *F* = 36*	SCC, clinically node negative	LRFS RR = 9.38%	Margin not significantly associated with local recurrence (*p* = 0.63)

### Patient characteristics

Our 150 patients displayed a mean age of 60.28 $\pm$ 16.39 years at the time of surgery (Table 2). We gathered the LS status of 149 patients, of which 13 (20.81%) were positive (Table 2). The HPV status was known for 81 patients, of which 20 patients (24.69%) were HPV-positive (Table 2). The mean follow-up time was 49.4 $\pm$ 31.2 months with a median of 50.6 months (Table 2).

**Table 2:** Cohort characteristics (Variables marked with an asterisk (*) are encoded as: 0 = No; 1 = Yes)

Variable	Mean	SD	Min	Max	*n*
Age at surgery [years]	60.28	16.39	21.00	91.00	
Tumor diameter [mm]	24.47	17.40	2.00	107.00	
Infiltration depth [mm]	6.16	5.80	0.55	35.00	
Removed lymph nodes (left)	6.53	4.73	0.00	22.00	
Removed lymph nodes (right)	5.93	4.57	0.00	23.00	
Last follow-up [months]	49.40	31.18	0.89	158.69	
Time to relapse [months]	19.82	20.30	1.94	101.62	40
Time to death [months]	50.20	44.15	5.72	154.71	12
Variables	0	1	2	3	NA
Lichen sclerosus (LS)*	118	31			1
Lichen planus*	145	3			2
Vulval intraepithelial neoplasia (VIN)*	140	8			2
HPV Status	61	20			69
T-Stage		128	22		
N-Stage	105	22	23		
R-Stage	146	4			
Grading		4	144	1	1
Multifocal*	131	19			
Urethra infiltration*	135	15			
Vagina infiltration*	143	7			
Clitoris infiltration*	114	36			
Anus sphincter infiltration*	148	2			
Radical vulvectomy*	127	23			
Partial vulvectomy*	34	116			
Local excision*	139	11			
Primary surgery only*	25	125			
Primary surgery then radiation*	134	16			
Primary surgery then radiation					
and chemotherapy*	143	7			
Additional resection*	134	16			

### Disease free survival

Of the 150 patients, 41 (27.33 %) experienced a relapse within a mean of 21.76 months. Our cohort achieved a mean follow-up time of 43.52 months. The DFS after 2 and 5 years was 82 % and 74.67 %, respectively.

### DFS subgroup analysis

**Fig. 1:**
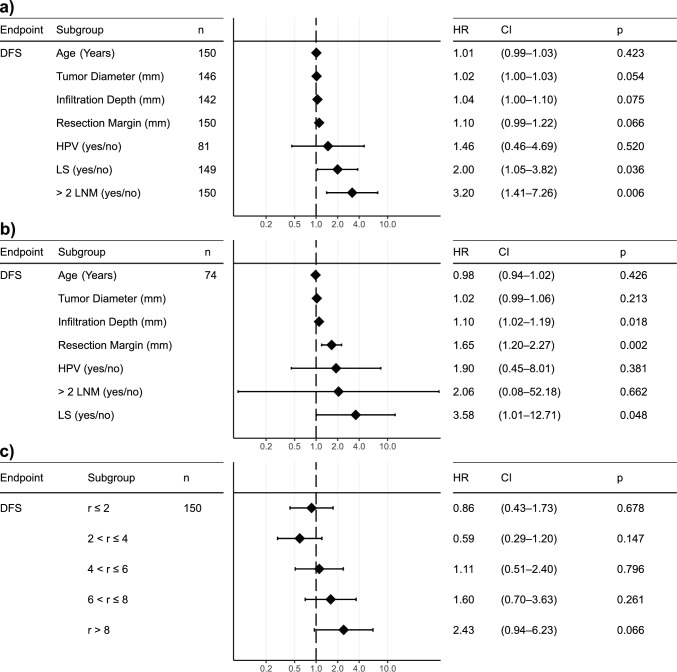
Forest plots **a** univariate analysis on disease-free survival (DFS) **b** multivariate analysis on DFS **c** DFS stratified by resection margin (in mm)

Univariate subgroup analysis demonstrated a hazard ratio (HR) of 2 for LS-positive patients (CI 1.05–3.82; *p *= 0.036) (Fig. 1a). Similarly, an HR of 3.2 (CI 1.41–7.26; *p *= 0.006) was observed for patients with two or more lymph node metastasis (LNM) (Fig. 1a). In a multivariate analysis, the influence of LNM on DFS was no longer significant due to a high variance in the variable (HR 2.06; CI 0.08–52.18; *p *= 0.662) (Fig. 1b). In contrast, the resection distance’s influence on DFS demonstrated an HR of 1.65, indicating shorter DFS in patients with wider resection margins (CI 1.20–2.27; *p *= 0.002) (Fig. 1b). LS remained significant in a multivariate setting (HR 3.58; CI 1.01–12.71; *p *= 0.048) (Fig. 1b). Infiltration depth was significantly associated with DFS in a multivariate analysis (HR 1.10; CI 1.02–1.19; *p *= 0.018) (Fig. 1b). HPV-positive patients showed a non-significant trend towards shorter DFS in both univariate (HR 1.46, CI 0.46–4.69, p=0.520) and multivariate analysis (HR 1.90, CI 0.45–8.01, *p *= 0.381) (Fig. 1a, b).

### Comparison of recurrence location

**Fig. 2:**
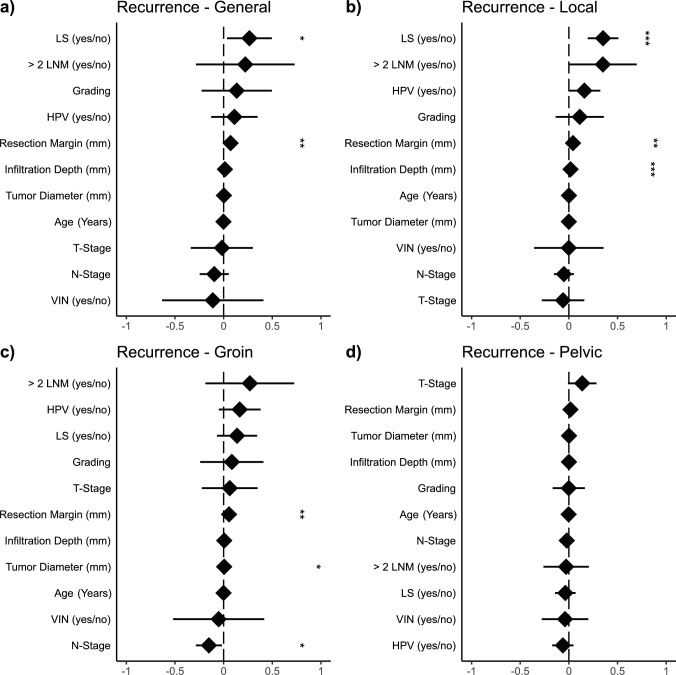
Fitted generalized linear models (GLM) based on different recurrence locations. (LNM = lymph node metastasis; *p < 0.05; **p < 0.01; ***p < 0.001)

To better understand the influence of our recorded parameters on the recurrence location, we fitted GLMs to general, local, groin, and pelvic recurrence (Fig. 2). For the general recurrence, both LS (*p *= 0.028) and resection distance (*p *= 0.003) displayed a significant influence (Fig. 2a). In contrast to this, the local recurrence GLM demonstrated a significant impact of LS (*p* $<$ 0.001), infiltration depth (*p* $<$ 0.001), and resection distance (*p *= 0.009) (Fig. 2b). Furthermore, resection distance significantly predicted groin recurrence (*p *= 0.010), as well as tumor diameter (*p *= 0.047) and N-Stage (*p *= 0.030) (Fig. 2c). Pelvic recurrence was not associated with any recorded parameters (Fig. 2d). Although a wider resection distance demonstrated a higher recurrence rate, the effect size in all three significant categories was minuscule (e.g., β = 0.055 for groin relapse).

### Resection distance

**Fig. 3:**
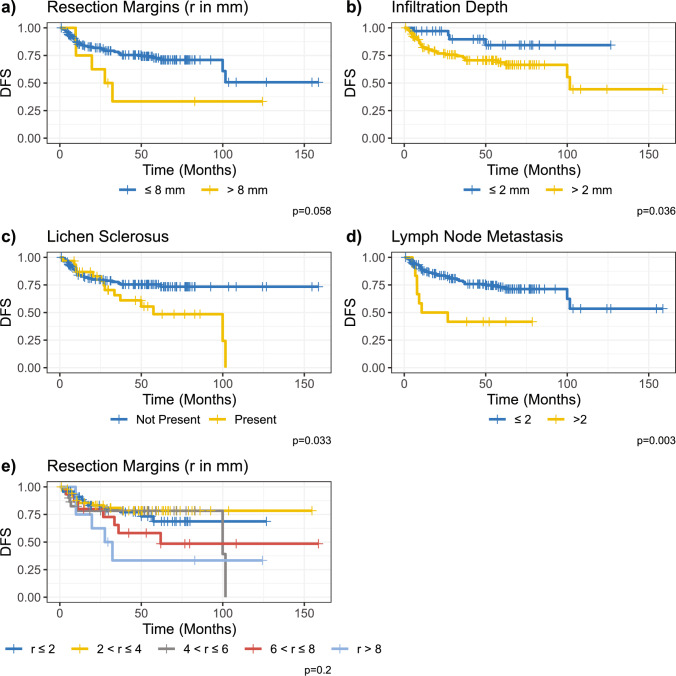
Kaplan–Meier analysis for **a** resection margins **b** infiltration depth of primary tumor **c** lichen sclerosus (LS) **d** lymph node metastasis **e** multiple resection cutoffs

To better understand the influence of the resection distance on the DFS, we performed a univariate Cox regression analysis with different levels of resection distance (Fig. 1c). The resection distance was divided into five levels: $r \le 2,\;2 < r \le 4\;4 < r \le 6\;6 < r \le 8\;{\text{and}}\;r> 8$ mm. No subgroup demonstrated a significant influence on HR. However, a non-significant trend towards a higher HR was observed, increasing with resection distance and a maximum HR of 2.4 for $r>8$ mm (CI 0.94–6.23; *p *= 0.066) (Fig. 1c). Similarly, this trend can be observed in the Kaplan–Meier analysis, while the log-rank analysis remains non-significant (*p *= 0.2) (Fig. 3e). Further, we performed a Kaplan–Meier analysis with only two groups: $r\le 8$ and $r>8$ mm, which demonstrated an almost significantly higher DFS in patients with lower resection distance (*p *= 0.058) (Fig. 3a).

### Further Kaplan–Meier analysis

Moreover, we performed a Kaplan–Meier analysis with different levels of infiltration depth (Fig. 3b). The patients were divided into two groups $\le 2$ and $>2$ mm. DFS was significantly higher in patients with an infiltration depth of $\le 2$ mm (p = 0.001) (Fig. 3b).

Similarly, patients with LS demonstrated inferior DFS in the Kaplan–Meier analysis (*p*=0.033) (Fig. 3c). Finally, the Kaplan–Meier analysis of lymph node metastasis demonstrated superior survival in patients with two or fewer metastatic lymph nodes (*p *= 0.003) (Fig. 3d).

## Discussion

This study's findings contribute to the ongoing debate regarding the optimal resection margin in the surgical management of vulvar SCC. Our results suggest that the traditionally recommended resection margin of > 8 mm may not be as critical for recurrence and survival as previously assumed. Instead, our data highlight the significant role of other factors, particularly the presence of LS and lymph node metastasis.

Our study aligns with the growing body of literature challenging the necessity of a resection margin greater than 8 mm. This finding is consistent with 12 of the 18 studies in our literature review, which found no significant association between resection margin distance and survival or recurrence.

The multifactorial nature of vulvar cancer recurrence may contribute to the observed lack of a strong correlation between resection margin distance and DFS. While adequate surgical margins are essential, other factors like tumor biology, lymph node involvement, and underlying conditions like LS appear to play more critical roles in patient outcomes. Our multivariate analysis further supports this; resection margin distance demonstrated a minimal impact on DFS, with an HR of 1.14 (CI 1.01–1.28; *p *= 0.029).

LS was significantly associated with a higher risk of recurrence and reduced DFS in univariate and multivariate analyses. This underscores the importance of LS as a prognostic factor in vulvar SCC. Our findings indicate that patients with LS may benefit from more intensive monitoring and adjunctive therapies, irrespective of the achieved resection margin. The importance of lifelong local potent cortisone application (like clobetasol 0.05%) 1–2/times per week, which is recommended in the current guidelines, is crucial and reduces the risk of recurrence [5, 26]. The significant association between LS and general and local recurrence, as identified in our GLM, further supports these recommendations.

Lymph node metastasis, notably when two or more nodes are involved, also emerged as a critical determinant of DFS. Our Kaplan–Meier analysis revealed a markedly inferior DFS in patients with more than two metastatic lymph nodes (*p *= 0.003), reinforcing the importance of comprehensive lymph node staging and, when appropriate, aggressive management of nodal disease with adjuvant radio-(chemo)therapy and close follow-up [27].

The findings from our study suggest that a one-size-fits-all approach to resection margins in vulvar SCC may not be appropriate. While the current NCCN guidelines recommend margins of 1–2 cm, our data suggest that a more nuanced approach, taking into account individual patient risk factors such as LS status and lymph node involvement, may be more beneficial. In cases where these risk factors are absent, especially in young patients or those where organ preservation (especially the clitoris) is a priority, smaller margins may be justified without significantly compromising DFS.

However, while our data support the potential for more conservative margins in certain patients, this approach requires careful patient selection and thorough preoperative counseling. The patients should be informed of the potential risks associated with reduced resection margins, and close postoperative follow-up is essential to identify and manage any local recurrence promptly.

This study is limited by its retrospective design and single-center setting, which may restrict the generalizability of the findings. Additionally, the small sample size, particularly in subgroup analyses, may reduce the power to detect significant differences in some comparisons.

## Supplementary Information

Below is the link to the electronic supplementary material.

Supplementary file1 (DOCX 465 kb)

## Data Availability

The data that support the findings of this study are available from the University Hospital Düsseldorf. De-identified data may be made available from the corresponding author upon reasonable request and with permission from the University Hospital Düsseldorf.

## Abbreviations

CIS: Carcinoma in situ
DFS: Disease free survival
GLM: Generalized linear models
HPV: Human papillomavirus
LS: Lichen sclerosus
NCCN: National Comprehensive Cancer Network
SCC: Squamous cell carcinoma
VIN: Vulval intraepithelial neoplasia
