# Supplementary material for: Influence of resection distance on vulvar cancer relapse: a retrospective analysis
Source: Arch Gynecol Obstet. 2025 May 21;312(2):611–20. doi: 10.1007/s00404-025-08046-y (PMC12334497; doi:10.1007/s00404-025-08046-y)

Influence of Resection Distance on Vulvar Cancer Relapse:
A Retrospective Analysis

Cieslik, Jan-Philipp^a,b*^; Beerbaum, Annika Sophia^a,b*;^

Fehm, Tanja^a,b^; Hampl, Monika^a,b^

^a^ Department of Gynecology and Obstetrics, University Hospital Düsseldorf, Germany

^b^ Center for Integrated Oncology (CIO Aachen, Bonn, Cologne, Duesseldorf), Germany

^*^ equal contribution

# Corresponding Author Information

Professor Monika Hampl
University Hospital Düsseldorf
Moorenstraße 5, 40225 Düsseldorf Germany
[Hampl@med.uni-duesseldorf.de](mailto:Hampl@med.uni-duesseldorf.de)

# Supplementary Material

**Table S1**: PubMed search terms for our literature review

| Query # | Query | Hits |
| --- | --- | --- |
| #5 | #1 AND #2 NOT #3 NOT #4 | 60 |
| #4 | "Paget" [Title] | 6,868 |
| #3 | "Review"[Title] OR "Review"[Publication Type] OR "systematic review"[Publication Type] OR "Meta-Analysis"[Publication Type] OR "case reports"[Publication Type] | 5,923,755 |
| #2 | ("Resection"[Title/Abstract] AND "Margin"[Title/Abstract]) OR ("Resection"[Title/Abstract] AND "Distance"[Title/Abstract]) OR ("Margin"[Title/Abstract] AND "Distance"[Title/Abstract]) | 22,342 |
| #1 | "Vulvar Cancer"[Title/Abstract] OR "Vulvar Neoplasms"[MeSH Terms] | 9,623 |

**Figure S1**: PRISMA flow chart from our literature review


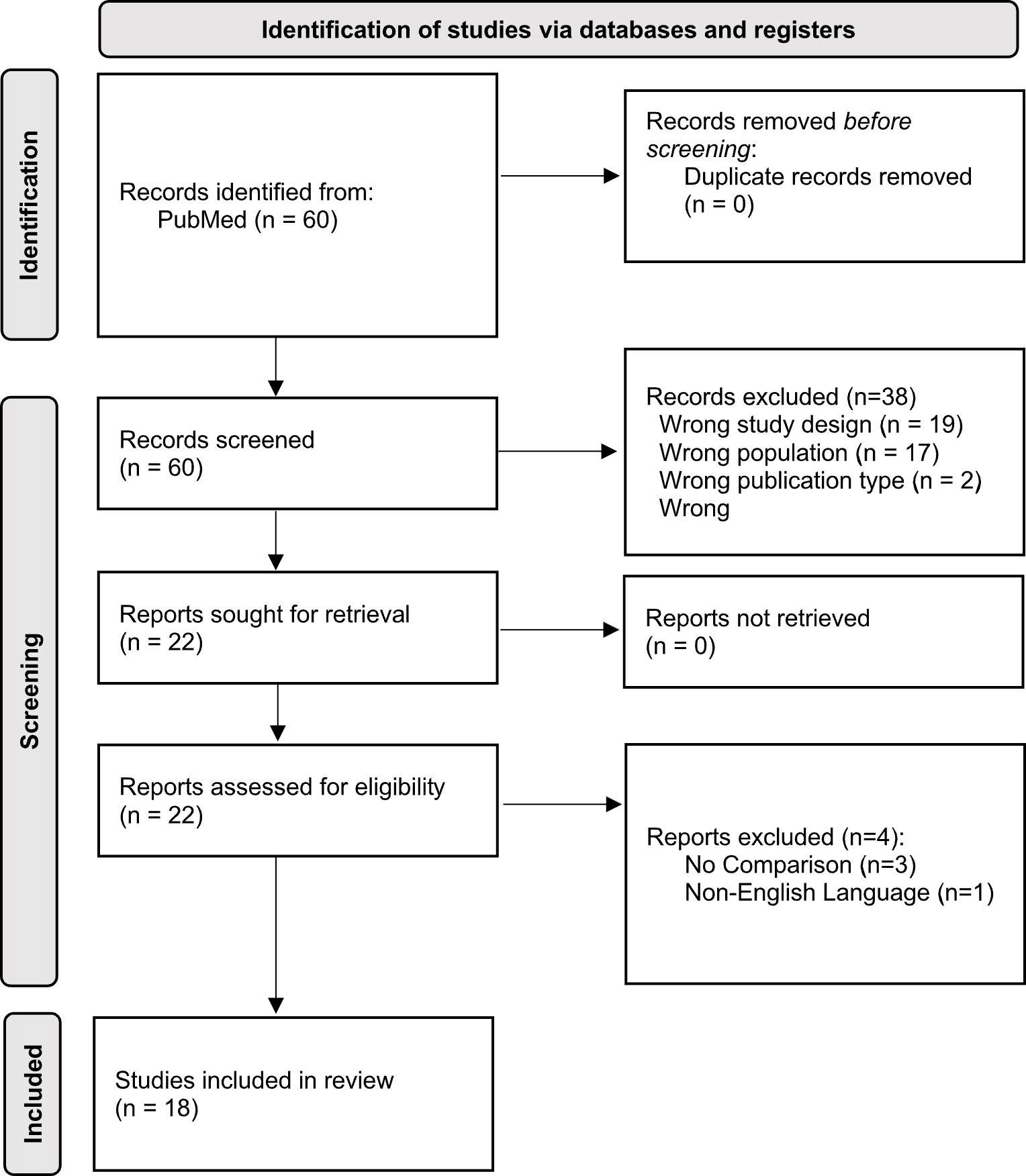

Supplement: Supplementary file 1 — Supplementary file1 (DOCX 465 kb) [file 404_2025_8046_MOESM1_ESM.docx]
